# Image Encryption Scheme Based on Orbital Shift Pixels Shuffling with ILM Chaotic System

**DOI:** 10.3390/e25050787

**Published:** 2023-05-12

**Authors:** Wajid Ali, Congxu Zhu, Rabia Latif, Muhammad Asim, Muhammad Usman Tariq

**Affiliations:** 1 School of Computer Science and Engineering, Central South University, Changsha 410083, China; wajid7230@gmail.com; 2 Artificial Intelligence and Data Analytics Laboratory, College of Computer and Information Sciences (CCIS), Prince Sultan University, Riyadh 11586, Saudi Arabia; rlatif@psu.edu.sa; 3 EIAS Data Science Lab, College of Computer and Information Sciences, Prince Sultan University, Riyadh 11586, Saudi Arabia; masim@psu.edu.sa; 4 School of Computer Science and Technology, Guangdong University of Technology, Guangzhou 510006, China; 5 Department of Marketing, Operations, and Information System, Abu Dhabi University, Abu Dhabi 13089, United Arab Emirates; muhammad.kazi@adu.ac.ae

**Keywords:** cryptosystem, chaos, ILM chaotic system, image encryption, orbital shift

## Abstract

Image encryption techniques protect private images from unauthorized access while they are being transmitted. Previously used confusion and diffusion processes are risky and time-consuming. Therefore, finding a solution to this problem has become necessary. In this paper, we propose a new image encryption scheme that combines the Intertwining Logistic Map (ILM) and Orbital Shift Pixels Shuffling Method (OSPSM). The proposed encryption scheme applies a technique for confusion inspired by the rotation of planets around their orbits. We linked the technique of changing the positions of planets around their orbits with the shuffling technique of pixels and combined it with chaotic sequences to disrupt the pixel positions of the plain image. First, randomly selected pixels from the outermost orbit are rotated to shift the pixels in that orbit, causing all pixels in that orbit to change their original position. This process is repeated for each orbit until all pixels have been shifted. This way, all pixels are randomly scrambled on their orbits. Later on, the scrambled pixels are converted into a 1D long vector. The cyclic shuffling is applied using the key generated by the ILM to a 1D long vector and reshaped into a 2D matrix. Then, the scrambled pixels are converted into a 1D long vector to apply cyclic shuffle using the key generated by the ILM. After that, the 1D long vector is converted into a 2D matrix. For the diffusion process, using ILM generates a mask image, which is then XORed with the transformed 2D matrix. Finally, a highly secure and unrecognizable ciphertext image is obtained. Experimental results, simulation analysis, security evaluation, and comparison with existing image encryption schemes show that it has a strong advantage in defending against common attacks, and the operating speed of this encryption scheme also performs excellently in practical image encryption applications.

## 1. Introduction

With the development of network and communication technology, multimedia has emerged as an essential tool for both personal and professional communication. Digital cameras are now widespread, and by providing access to everyone, the generation of digital images becomes easy. These images are present in almost every aspect of human life, such as family images, party images, social interaction images, biological images, medical images, satellite images, military images, and commercial images. Among these images, some are very sensitive and confidential. Apart from that, the transmission of these images over open-access networks, such as the Internet, is risky [1] since hackers seek such kinds of activities. Any information exchanged online needs strong security to prevent unauthorized access [2]. An image can contain a wealth of information beyond just the visual content of the image itself. For example, a personal image may include the identity of the person, their physical appearance, age, and location at the time the image was taken. 

For decades, researchers in cryptography have been developing various techniques to secure communication. They face a lot of challenges in developing techniques that can stand attacks and can be applicable in real-life situations. To present a highly secure algorithm, they used new algorithms with advanced mathematical models. One of the most well-known technologies for ensuring the security, veracity, and secrecy of images that are floating around the internet is the technique of image encryption and decryption [3,4]. 

Cryptographers have designed several historical ciphers such as Data Encryption Standard (DES), Advanced Encryption Standard (AES), and Rivest-Shamir-Adleman (RSA). DES and AES are the symmetric key algorithms used for data encryption. RSA is an asymmetric key method used for digital signature verification and encryption. However, these ciphers cannot be used to encrypt digital images due to their limitation in processing only text data [5,6,7]. Depending on the digital image’s size, it contains a lot of information having correlation pixels bounding as compared to the text data. The basic image’s significant association between adjacent pixels makes it difficult to eliminate [7,8].

At the beginning of 1989, Matthews developed a chaotic encryption technique to solve such kinds of problems [9]. The chaotic system demonstrates several distinctive characteristics, including ergodicity sensitivity to beginning conditions and complex nonlinear dynamics [10,11]. Habutsu et al. proposed the first chaotic stream cipher approach for image encryption in 1991 [12]. Later on, various chaotic image encryption algorithms were proposed [2,3,5,7,13,14,15,16,17,18,19,20,21,22,23,24,25,26,27,28,29,30,31,32,33,34,35,36,37]. To achieve a significant security improvement, some researchers have integrated these two processes into one stage [19,31,38,39,40].

According to a recent study, more than 32% of currently in-use image encryption algorithms are based on the chaotic theory [25,28,29,30,31]. For instance, in [19], chaos is used in a brand-new lifting transform-based image encryption technique. Using a 3D chaotic map and image pixel shuffle, authors of [20] suggested a novel image encryption method. They first used pixel shuffling to encrypt a plain image, after which the output was XORed with a key before being subjected to the 3D chaotic map. The image is permuted using a 3D logistic chaotic map proposed in [21]. Following permutation, the DNA rules are used to encode the pixel values, and another random sequence is used for DNA operations to form the encoded image [36,41]. In [34,42], block-based diffusion is proposed by using the crossover method to the pixel values and the XOR, XNOR, and random functions to carry out the block-based diffusion. A differential resistance attack is possible with this strategy. In [22], the modified ZigZag Transform (ZT) technique is used that exchanges the upper left and next horizontal neighboring pixels with the base right pixel for enhanced security. The modification is applied to every matrix in the image, starting from the upper left corner to the base right corner. The remaining elements of the matrix are swapped in a zigzag pattern to execute encryption. Researchers placed a high focus on speedy product delivery in addition to security considerations. Therefore, they turned to various strategies, including the swapping method, circular shift operations, interchanging method, block methods, and so on. Other image encryption methods are proposed as a result of the possibility that blocks may be formed [22,23,25,34,42,43,44].

Permutation-substitution architecture is the foundation for most of the methods that are provided. While substitution means the changing of pixel values of the shuffled images using the random sequence, permutation entails shifting the pixel position of an original image across a chaotic map. The chaos-based image encryption technique with a permutation and diffusion structure is proposed by Zhang et al. [17]. To solve the shortcomings of several image encryption methods based on the complete shuffle strategy, they developed two directional diffusion techniques. First, they diffuse in the forward direction, and later, they diffuse in the reverse way. They provided a rapid method to produce a permutation by merging some little permutations, where some permutations are formed directly by the chaos system. To create the large permutation required at the permutation stage, it takes some time to effectively implement this technique for encrypting high-quality images in real-life scenarios. A color image encryption technique is proposed by Zhang et al. [45]. They use a 2D chaotic cat map with another logistic map technique. In this proposed method, they changed the position of an image pixel by switching places with other pixels in a matched plane. Their method was speedier than the conventional method of shifting pixels inside a single plane in terms of cryptography. In [25], authors introduced the block cipher-based image encryption method. They used some other modes of operation, such as the counter mode and cipher block chaining, as well as chaotic sequences. An image encryption system has been proposed using the ideas of pixel swapping, masking, and permutation [25,27,34]. Some of them used methods that used eight and sixteen sequences for swapping, masking, and pixel permutation, respectively. The authors of this research broke the link between image pixels by randomly swapping them, taking advantage of permutation, and switching sequences. Finally, they performed the XOR operation using the masking sequence. In [35], the random scan pair and 2D standard map were used for pixel shuffle. In [25], the permutation and diffusion were performed at the block level as opposed to the pixel level to preserve time complexity. As a result of the full blocks switching rather than the pixels switching, the cipher’s security was breached. To improve the diffusion with reduced time complexity, we proposed a bit-level scrambling technique to achieve diffusion, which involves permuting the pixels of the image in a non-linear manner. This process effectively destroys any intra-correlations among the pixels of the image, which helps to enhance the security of the encryption. Furthermore, the key generation method presented in this work using ILM is not solely dependent on the image itself but also on certain secret parameters. This highlights the importance of maintaining the confidentiality of these secret parameters to ensure the robustness and effectiveness of our proposed scheme.

The main contribution of this paper includes the following stages to secure the image encryption process.

1. Inspired by the orbits and planets, we have designed a new encryption scheme. In the scheme, scrambling of the pixels has been linked to the movement of the planets in their orbits. This design makes the new cipher more secure and very uncorrected.
2. Multi level-round scrambling approaches are used to increase the cipher’s unpredictability and decrease the correlation between the pixels. The intertwining logistic map is used for key generation and masking of the scrambled temporary ciphertext image.
3. The purposed encryption technique is a secured encryption scheme that takes a very short execution time as compared to existing schemes and passes all the security tests.
4. Machine experimentation provided very promising results which preponderate those of many of the published works.

The remaining content of this paper is organized as follows: Section 2 introduces the Preliminaries part. The core idea of the proposed technique is described in Section 3. In Section 4, we present some simulation results and security analysis. Finally, in Section 5, the proposed scheme is summarized and concluded.

## 2. Preliminaries

In this section, we discuss the preliminaries which are added to our proposed method in an intertwining logistic map.

### The ILM Chaotic System

The chaos theory studies systems that are highly dynamic and sensitive to two main components: the system parameters and the initial values of the system or map. In our proposed methodology, we use the intertwining logistic map (ILM) [27].

(1) $$\left\{\begin{array}{c} x_{i+1}=\left[\mu \times k_{1}\times y_{i}\times \left(1-x_{i}\right)+z_{i}\right]mod\;1\\ y_{i+1}=\left[\mu \times k_{2}\times y_{i}+z_{i}\times \frac{1}{1+x_{i+1}^{2}}\right]mod\;1\\ z_{i+1}=[u\times \left(x_{i+1}+y_{i+1}\times k_{3})\times Sinz_{i}\right]mod\;1 \end{array}\right.$$

**Figure 1:**
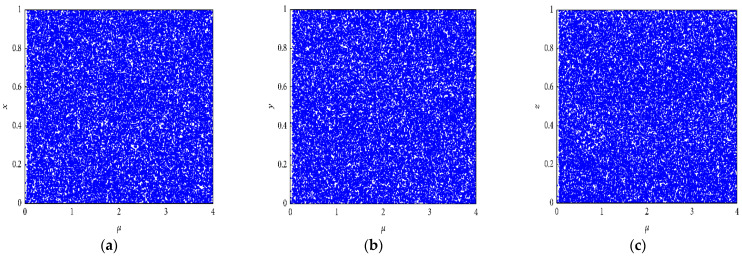
Bifurcation diagrams of the (ILM). (**a**) for *x*, (**b**) for *y*, and (**c**) for *z*.

According to the given equation, when 0 < *μ* ≤ 3.999, |*k*_1_| > 33.500, |*k*_2_| > 37.970, and |*k*_3_| > 35.700, system (1) is chaotic. With these parameters and certain initial values, the above-mentioned equations show that ILM generates three chaotic streams of (*x*, *y*, *z*). Figure 1 shows the bifurcation diagrams of the intertwining logistic map for $k_{1}=31.8809$, $k_{2}=31.891$, and $k_{3}=35.318$. From Figure 1, it can be seen that the ILM is more chaotic and lacks any open spaces compared to a logistic map. Therefore, the intertwining logistic map is more suitable for image encryption than a logistic map.

## 3. Proposed Methodology

The proposed encryption method applies the OSPSM technique to confuse image pixels. This method draws inspiration from the rotational motion of planets along their orbits. We linked the technique of changing the positions of planets around their orbits with the shuffling technique of pixels and combined it with chaotic sequences to disrupt the pixel positions of the image. The encryption scheme consists of three main steps. The first step is the orbital shift shuffling. In this step, the pixels of a standard image are extracted into a 2D matrix. The orbital shift shuffling technique is then applied to the matrix to shuffle the pixel positions randomly. For instance, to apply OSPSM, the pixel values of plain images are stored in a 2D array that represents the image height and image width. According to the key, pixels from the orbits are randomly selected, and a rotational shift of these pixels is initiated, causing all pixels on the orbits to change their original positions due to the shifting of the selected pixels. This process continues until the pixels of the innermost orbit have completed shifting. Pixel shifting is the process of changing a pixel’s original position based on a secret key generated by ILM. Through this method, the positions of all pixels in the orbit are randomly shuffled. The second step is the cyclic scrambling of a 1D vector. After the orbital shift, the pixel matrix is converted into a 1D long vector. The key generated by ILM is then used to cyclically shuffle the elements of the vector. Finally, the scrambled 1D long vector is converted back into a 2D matrix. The third step is the pixel value transformation based on chaotic mask images. In this step, the diffusion operation is carried out on the scrambled 2D matrix, which involves two steps. The first step is to generate a mask image using ILM. Next, perform the XOR operation between the generated mask image and the scrambled 2D image matrix. Finally, the ciphertext image is obtained.

### 3.1. Key Generation Method

The proposed method uses an intertwining logistic map. The input for encryption is a 256 × 256 grayscale image. The starting keys for the intertwining logistic map are determined as follows:

(2) $$x_{0}=x_{0}^{\prime }+\frac{\sum_{i=1}^{64}\sum_{j=1}^{256}I_{i\times j}}{16384\times 128}$$

(3) $$y_{0}=y_{0}^{\prime }+\frac{\sum_{i=65}^{128}\sum_{j=1}^{256}I_{i\times j}}{16384\times 128}$$

(4) $$z_{0}=z_{0}^{\prime }+\frac{\sum_{i=129}^{192}\sum_{j=1}^{256}I_{i\times j}}{16384\times 128}$$

(5) $$\mu =\mu^{\prime }+\frac{\sum_{i=193}^{256}\sum_{j=1}^{256}I_{i\times j}}{16384\times 128}$$

(6) $$k_{1}=k_{1}^{\prime }+\frac{\sum_{i=1}^{128}\sum_{j=1}^{256}I_{i\times j}}{16384\times 256}$$

(7) $$k_{2}=k_{2}^{\prime }+\frac{\sum_{i=129}^{256}\sum_{j=1}^{256}I_{i\times j}}{32768\times 256}$$

(8) $$k_{3}=k_{3}^{\prime }+\frac{\sum_{i=1}^{256}\sum_{j=1}^{256}I_{i\times j}}{65536\times 256}$$

where “*I*” represents the plain image and (*i*,*j*) are the indices of the input plain image, the variables $x_{0}^{\prime },y_{0}^{\prime },z_{0}^{\prime },\mu^{\prime },k_{1}^{\prime },k_{2}^{\prime }$, and $k_{3}^{\prime }$ in Equations (2)–(8) are the variables of the ILM before embedding the plain text sensitivity. The variables $x_{0},y_{0},z_{0},\mu ,k_{1},k_{2}$, and $k_{3}$ are the variables of the ILM plain text sensitivity after embedding.

### 3.2. Scrambling Methods

**Figure 2:**
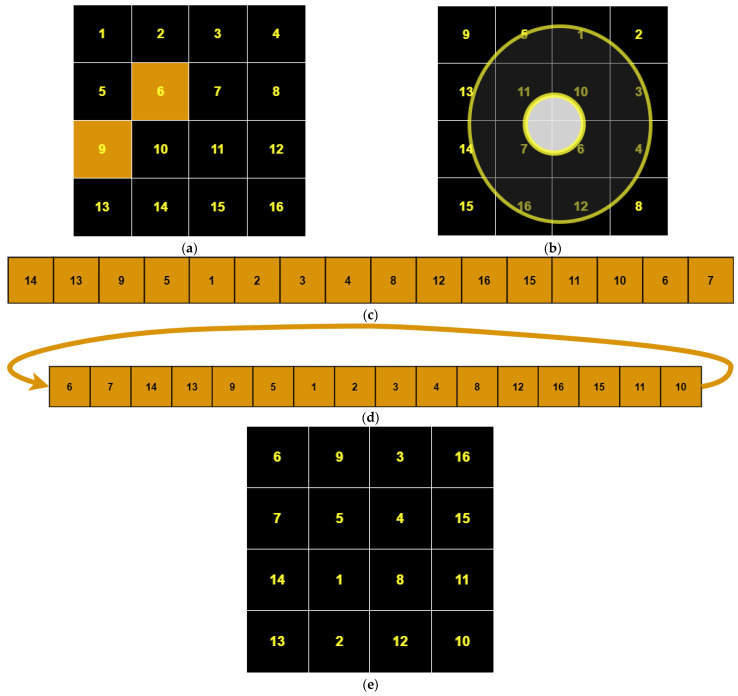
The schematic diagram of OSPSM transformation. (**a**) plain matrix image, (**b**) orbital shuffle image, (**c**) 1-D long vector image, (**d**) cyclic shuffle image, (**e**) 2-D image matrix.

OSPSM (Orbital Shift Pixels Shuffling Method) is a newly proposed scrambling method for digital images. It operates by scanning the pixel values in an orbital form. First, it shuffles all the pixel values of the outermost orbit, starting from the position of the randomly selected pixel (*r*, *c*), and then goes to the second outer orbit pixels to shuffle the elements of the second outer orbit starting from the randomly selected pixel position (*r’*, *c’*), and so on, until it completes all the orbits of the original image according to the size of the image. We elaborated OSPSM with an example shown in Figure 2, considering a matrix size of 4 × 4 as shown in Figure 2a. In Figure 2b, the pixels of orbits are shuffled by applying OSPSM, starting from the second to last element of the outermost orbit and shuffling all the pixels of the outermost orbit according to the key values. Then, the inner orbit pixels are shuffled, and after the completion of the orbit pixels shuffling, all the pixel values are stored in a long vector form shown in Figure 2c. Later on, the vector is randomly permutated by applying cyclic shuffling techniques using the key value $k\in \left(-2^{256},2^{256}\right)$ shown in Figure 2d, then reshaped into a 2D matrix shown in Figure 2e. The image that has been scrambled can then be obtained. 

**Figure 3:**
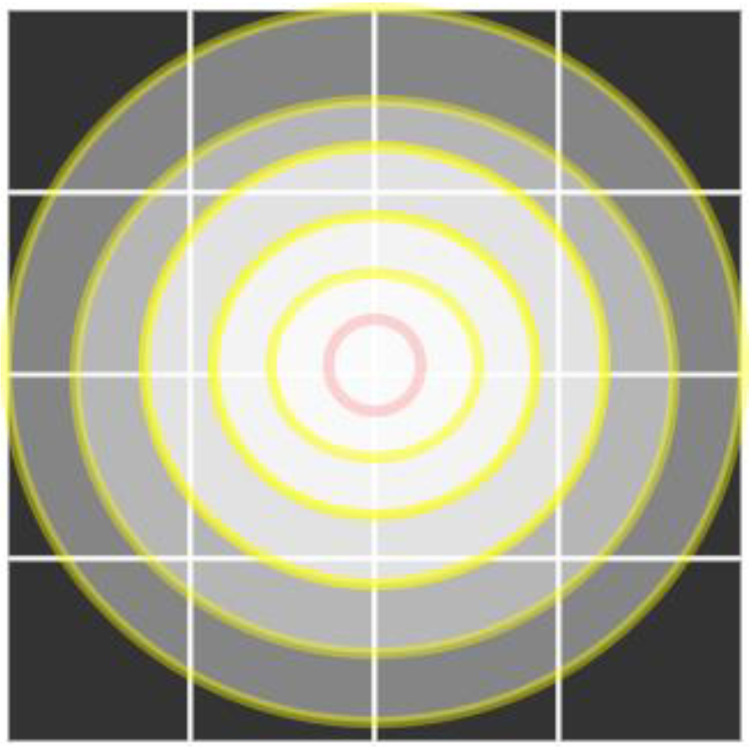
OSPSM transformation general schematic diagram.

Figure 3 shows the general schematic diagram of OSPSM transformation. Similarly, we generalize the OSPSM transformation of an example of a 4 × 4 matrix to the size of the *m* × *m* matrix. When we read the image of size *m* × *m*, the turning point of the transformation appears in a round shape which is just like an orbit’s shape transformation in the middle of the image. If *m* is even, *m*/2 orbits, and for odd, (*m* − 1)/2 orbits will be constructed. Step by step procedure of this diagram is shown below to understand the general permutation method of the proposed scheme. 

**Figure 4:**
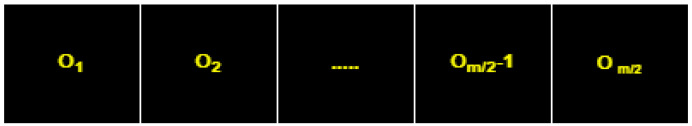
1D long vector.

**Figure 5:**
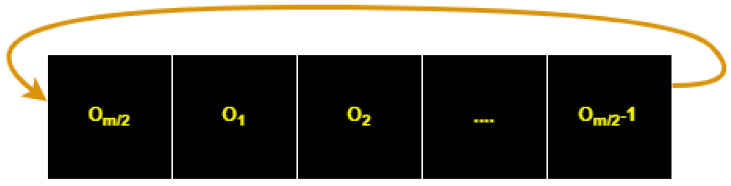
Cyclic permutation long vector.

After performing the OSPSM on the plain image, we converted the scrambled image into one long vector in the dimension 1×m×m, which is shown in Figure 4. The circular permutation is applied to the long vector, which is shown in Figure 5. 

### 3.3. Encryption Scheme

Many scrambling and encryption methods use basic operations called permutation transformations. The chaos-based image cryptosystem has two iterative stages. Typically, the permutation stage provides the confusion effect, while the pixel value diffusion stage produces the diffusion effect. The relationship between the key and the ciphertext is maintained as complicated as possible by confusion. The image’s pixels are permuted during the confusion stage without having their values altered. At the diffusion step, the pixel values are altered consecutively, making a minor change to one image pixel results in a significant change to the entire image. In the confusion stage, the permutation of pixels is introduced to decorrelate the relationship between neighboring pixels.

**Figure 6:**
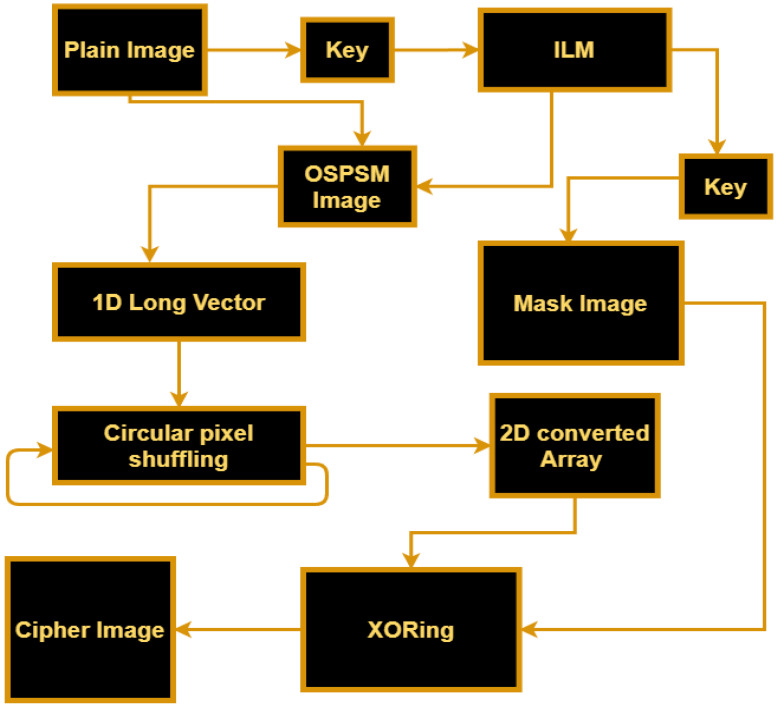
The block diagram of the proposed encryption scheme.

We proposed an image encryption technique based on OSPSM and ILM. Firstly, the initial keys of the chaotic map are generated using pixels of the plain image to make the scheme plain text sensitive. Secondly, the intertwining logistic map is generated using keys obtained in the first step, and the first sequences of the map are utilized as a key value for permutations. The positions of the pixels are shuffled over the entire image without any changing of the value of pixels, which means that the pixels of the plain image go to pixel-level scrambled using OSPSM. After applying OSPSM, the scrambled image is converted into a long vector. Thirdly, we have applied a cyclic shift operation on the vector obtained in the second step to obtain more distortion among the pixels. In the last step, a masked image is generated by intertwining logistic map. The generated mask image is then XORed with the scrambled two-dimensional image matrix. The XORing operation is used to modify the values of the image pixels to make our strategy resistant to histogram attacks. This cipher image is more secure as compared to the other proposed methods. The block diagram of our proposed encryption scheme is shown in Figure 6, which will help to understand the proposed method easily.

The following is a description of the necessary actions in our suggested plan.

Step 1: Take the original image as $I_{m*m}$ and generate keys of the chaotic map by using the pixels of the original image, the method is discussed in Section 3.

Step 2: Through the intertwining logistic map, the sequences $\left\{x\right\}$, $\left\{y\right\}$, and $\left\{z\right\}$ are generated m×m times. These sequences of real numbers are converted into integer numbers as follows:

(9) $$S_{i}^{1}=round\left(mod\left(\left|x_{i}\right|\times 10^{16},256\right)\right)+1,$$

(10) $$S_{i}^{2}=round\left(mod\left(\left|y_{i}\right|\times 10^{16},65,535\right)\right)+1,$$

(11) $$S_{i}^{3}=round\left(mod\left(\left|z_{i}\right|\times 10^{16},256\right)\right)+1,$$

where i=1,2,…,mm, and $S_{i}^{1}$, $S_{i}^{2}$, $S_{i}^{3}$ are the sequences of integer numbers. Original image pixels are scrambled with OSPSM and the key obtained by permutation is from the sequence $S_{i}^{1}$. Then we obtained the scrambled image as $I_{s}$.

Step 3: After performing with the OSPSM on the plain image, the scrambled image is converted into one long vector $I_{sv}$ in one dimension 1×m×m. The circular permutation is applied to this vector. The vector is permutated using the key value k from $S_{i}^{2}$ and then reshaped into a two-dimensional (2D) matrix $I_{svm}$.

Step 4: In this step, the diffusion operation is performed. For diffusion, a mask image is constructed by converting $S_{i}^{3}$ into matrix M, having the dimension of the image. After obtaining the mask image M, the XORing operation takes place between the image pixels and corresponding matrix values as:

(12) $$Cipher_{image}=I_{svm}(i,j)⊕M\left(i,j\right)$$

Finally, we obtained the cipher image.

In the field of cryptography, there are two methods for encryption, one is a private key and the second one is a public key. Our approach in this work is the private key method. Therefore, the decryption procedure has been carried out in the opposite order of the encryption algorithm.

## 4. Simulation Results and Security Analysis

In cryptography, different types of attacks can occur, such as differential attacks, noise and entropy attacks, selected plaintext/ciphertext attacks, and brute force attacks. An image encryption method should have enough strength to prevent such kinds of attacks. In this method, four grayscale images (Lena, a moon, an airplane, and a clock) with a size of 256 × 256 are used to show the effectiveness of the proposed method. 

In this work, the hardware and software settings that are used during the implementation of our proposed scheme are Intel^®^ core™ i3-55500U CPU@ 2.10 GHz, 16 GB RAM, 512 GB SSD. Window 10Pro, MATLAB R2021b.

### 4.1. Validation of Encryption

**Figure 7:**
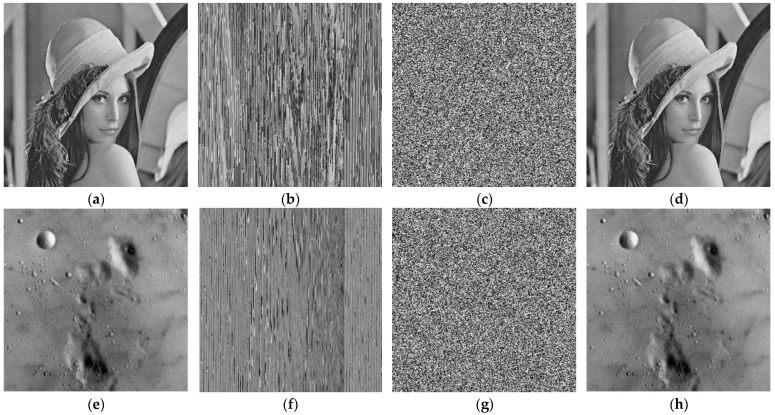

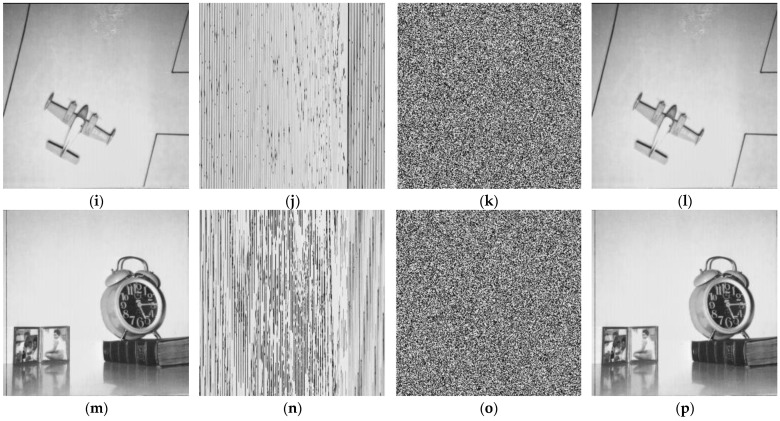
Results of the proposed image encryption scheme. (**a**) plain image of Lena, (**b**) shuffled image of Lena, (**c**) encrypted image of Lena, (**d**) decrypted image of Lena, (**e**) plain image of moon, (**f**) shuffled image of moon, (**g**) encrypted image of moon, (**h**) decrypted image of moon, (**i**) plain image of Airplane, (**j**) shuffled image of Airplane, (**k**) encrypted image of Airplane, (**l**) decrypted image of Airplane, (**m**) plain image of clock, (**n**) shuffled image of clock, (**o**) encrypted image of clock, (**p**) decrypted image of clock.

We examined our proposed method with some images to verify its better performance. The encryption and decryption effects are shown in Figure 7. Figure 7a,e,i,m are the plain images with a size of 256 × 256. Figure 7b,f,j,n are pixels shuffling images. Figure 7c,g,k,o are the encrypted images. Figure 7d,h,l,p show the decrypted images.

### 4.2. Key Space Analysis

Key space is an essential consideration for developing certain cipher images. If the key space is small, a possible adversary’s brute force attack can successfully break it. It should therefore be sufficiently large to prevent any such attack from succeeding on time. Sometimes, an adversary generates every key conceivable and tries to recover the original image. In this method, the encryption key consists of the listed initial values (x,y,z) and parameters $(\mu ,k_{1},k_{2}{,k}_{3}).$ The key value for a circular shift is taken from $S^{2}$ which is $2^{256}$. Moreover, if the computation precision is $10^{-15}$, this then contributes ${\left(10^{15}\right)}^{7}=10^{105}$ to the key space. Therefore, the overall key space comes out as $2^{256}\times 10^{105}=1.16\times 10^{182}$. Thus, it can be said that the proposed work is superior to some of the earlier ones in terms of the key space. Table 1 shows the key space of our proposed scheme and compares it to other existing methods.

### 4.3. Key Sensitivity Analysis

**Figure 8:**
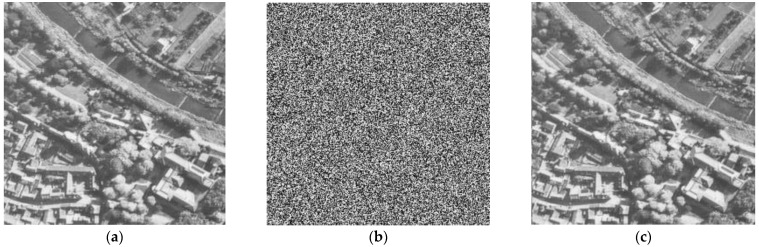
Encryption/Decryption with the original key. (**a**) plain image Aerial, (**b**) encrypted image of Aerial, (**c**) decrypted image of Aerial.

The key should be sensitive and have a big key size for a strong barrier to all techniques of brute force assaults to provide secure encryption. The original secret key was changed slightly for the testing of the sensitivity of the key. Our proposed method key contains external parameters, as shown in Table 2, which also shows the keys used in the proposed method by using the original keys. Consequently, we obtain the plain image after encryption and decryption using the original key parameters without any modifications, as shown in Figure 8. 

**Figure 9:**
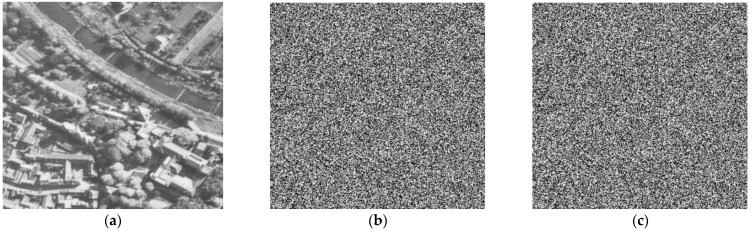
Encryption/Decryption with the slight change of key. (**a**) plain image Aerial, (**b**) slight change encrypted image of Aerial, (**c**) decrypted image of Aerial.

To test the key sensitivity, we made slight changes to the initial key and evaluated the effects. Specifically, it is not possible to obtain the plain image with a slight change in the key, as shown in Figure 9. Table 3 presents the change rates between two encrypted images assembled using the initial key, ${Key}_{1}$ (as shown in Table 2) and ${Key}_{n}$ (where *n* = 1, 2, …, 7), with slight variations in the initial keys ($x_{o}$, $y_{o},$ $z_{o},$ *μ*, $k_{1}$, $k_{2}$, $k_{3}$). The resulting NPCR (number of pixel changing rate, NPCR) for each pair of encrypted images is greater than 99.59%, indicating a high sensitivity to key changes. 

Therefore, we can conclude that the suggested encryption technique is highly sensitive to irregular keys since we have demonstrated in Figure 8 and Figure 9 that it is only possible to obtain the plain image after encryption and decryption using the original key parameters without any modifications. Thus, the key sensitivity of this algorithm achieves the expected effect.

### 4.4. Histogram Analysis

**Figure 10:**
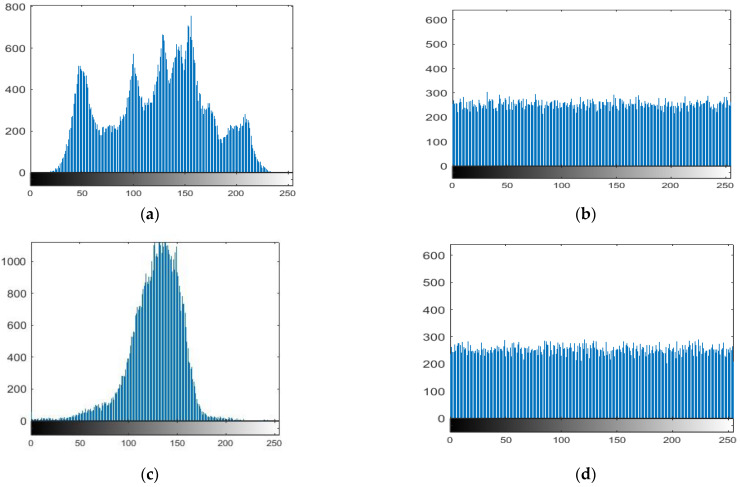

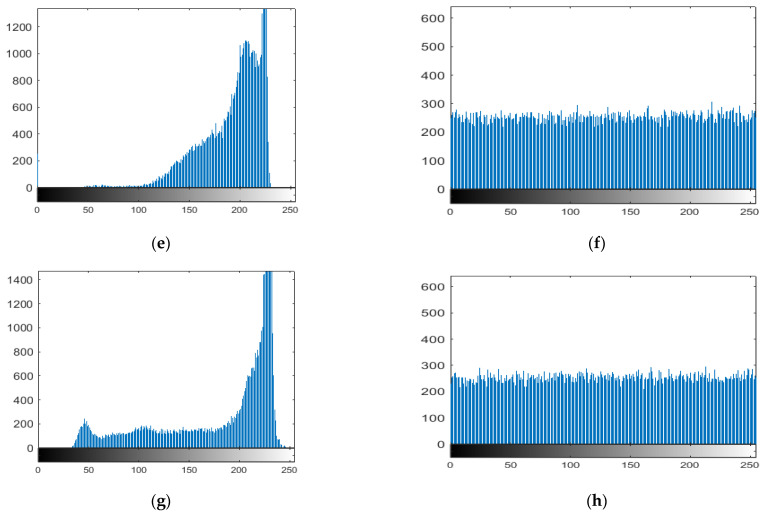
Histogram of the plain images and corresponding cipher images. (**a**) histogram of plaintex Lena, (**b**) histogram of ciphertext Lena, (**c**) histogram of plaintex moon, (**d**) histogram of ciphertext moon, (**e**) histogram of plaintex airplane, (**f**) histogram of ciphertext airplane, (**g**) histogram of plaintex clock, (**h**) histogram of ciphertext clock.

A graphic representation of data regarding the distribution of pixel values is an image’s histogram. The histogram of an ideal encrypted image should be equally distributed and completely different from a plain image to prevent competitors from deriving any pertinent information from the streaming histogram of an encrypted image. We conducted a statistical analysis, and the findings are presented below to demonstrate that our suggested approach is difficult to hack. Figure 10a,c,e,g demonstrates the histograms of the original images (Lena, moon, plane, and clock) with their cipher image histogram in Figure 10b,d,f,h. The size of the original images is 256 × 256.

### 4.5. Correlation Analysis 

**Figure 11:**
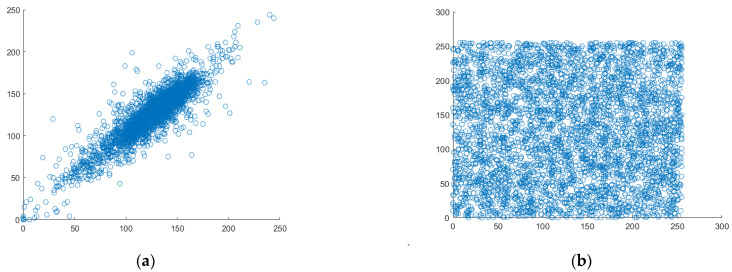
Analyzing the correlation between horizontal pixel pairs. (**a**) Horizontal correlation in plaintext, (**b**) Horizontal correlation in ciphertext.

**Figure 12:**
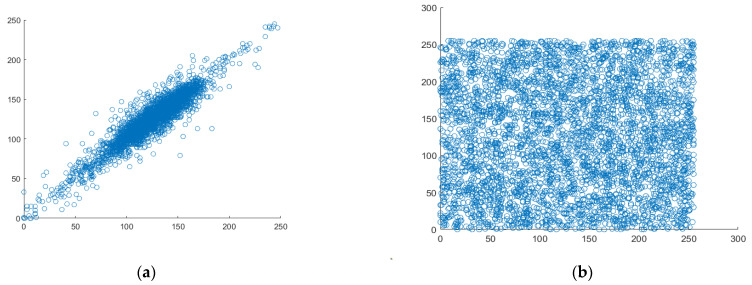
Analyzing the correlation between vertical pixel pairs. (**a**) vertical correlation in plaintext, (**b**) vertical correlation in ciphertext.

**Figure 13:**
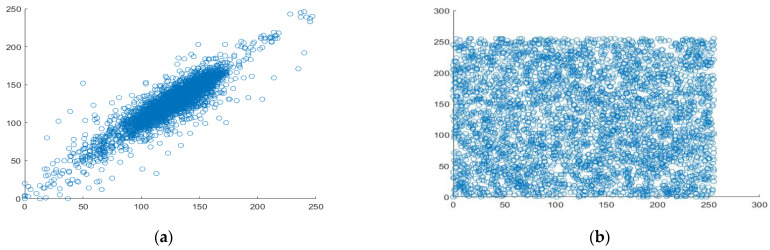
Analyzing the correlation between diagonal pixel pairs. (**a**) diagonal correlation in plaintext, (**b**) diagonal correlation in ciphertext.

The most crucial quality of data that belongs to the category of digital images is good correlation. Each pixel is highly correlated with the pixels around it. Therefore, it could be situated horizontally, vertically, or diagonally. Figure 11, Figure 12 and Figure 13 display scatter plots that indicate the connection between 4000 randomly chosen pixels in different directions, such as horizontal, vertical, and diagonal of both the original plain image and the created cipher image. For the correlation testing, a grayscale moon image with a size of 256 × 256 is used.

### 4.6. Information Entropy 

Entropy, commonly referred to as Shannon’s entropy, is one of the common measures used to assess the strength of a symmetric cryptosystem. Equation (12) is used to determine the entropy value.

(13) $$H\left(m\right)=-\sum_{i=0}^{N-1}P\left(m_{i}\right){log}_{2}\left[P\left(m_{i}\right)\right]$$

where *N* denotes the gray level of the image, *m_i_* denotes the symbol’s source, and last *P*(*m_i_*) denotes the symbol’s probability. The entropy values of some standard test images, such as Lena, a moon, a plane, a clock, etc., are demonstrated in Table 4. The ideal entropy value is 8.0. Table 5 proves that the entropy values of the encrypted images of the proposed method are nearest to the ideal value as compared to the existing methods.

### 4.7. NPCR and UACI

NPCR rate and UACI are two important measures of image encryption that we use. These values are used to assess how to resist the image encryption method by differential attacks. The algorithm’s sensitivity to changes in the plain image is evaluated using NPCR.

The values of NPCR and UACI are determined using the equations below.

(14) $$NPCR=\sum_{i,j}\frac{D\left(i,j\right)}{MN}\times 100\%$$

as we know that

(15) $$D\left(i,j\right)=\left\{\begin{array}{c} 0,if\;C1\left(i,j\right)=C\left(i,j\right)\\ 1,if\;C1\left(i,j\right)\neq C\left(i,j\right) \end{array}\right.$$

The averaged changing intensity of two separate decrypted images of an object is tested using UACI, which can be calculated by Equation (15).

(16) $$UACI=\sum \frac{\left|C1\left(i,j\right)-C2\left(i,j\right)\right|}{M\times N\times 255}\times 100\%$$

In the above equation, *C*1 and *C*2 are two unique cipher images made using two unique keys. Table 6, demenostarte the NPCR and UACI values represent the superior performance of our proposed method. Table 7 demenostarte the comparsion values of NPCR and UACI with existing methods

### 4.8. Analysis of Noise

**Figure 14:**
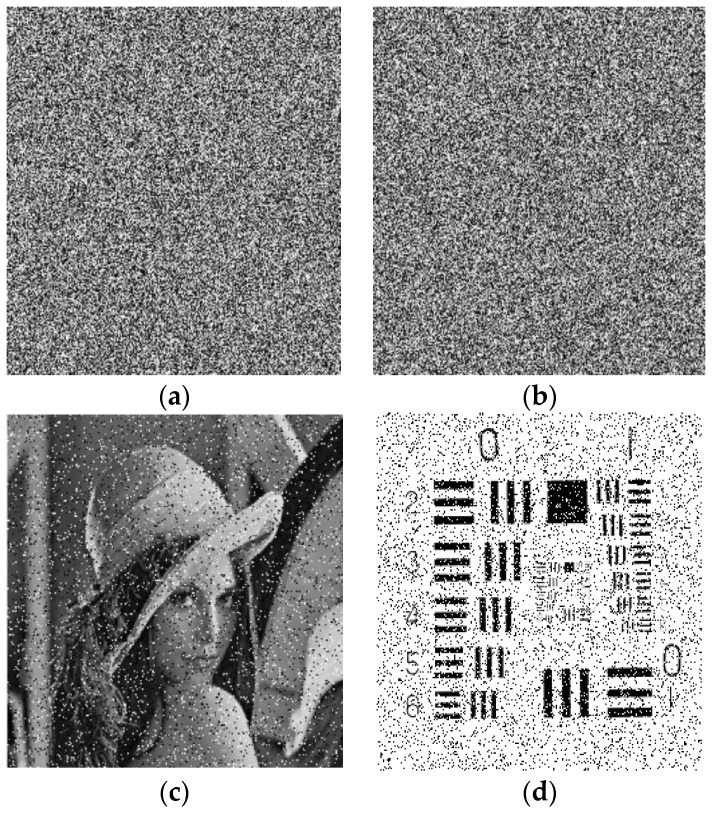
Attack test with different noise densities of Lena and Resolution Chart image. (**a**) noise density of 0.1 Lena encrypted image, (**b**) noise density of 0.3 resolution chart encrypted image, (**c**) noise density of 0.1 Lena decrypted image, (**d**) noise density of 0.3 resolution chart decrypted image.

In a real-life situation, the transmission of images may suffer from contamination due to some kind of noise, and occasionally, some part of the image during transmission is also lost. The anti-noise ability is very important for an image encryption algorithm. A good encryption scheme is assumed to defeat noise attacks as well. In the experiment, we introduced the salt and pepper noise with noise densities of 0.1 and 0.3 to the encryption image of Lena and a baboon. Figure 14 shows the encrypted and decrypted images of Lena and 5.1.13, polluted by salt and pepper noise, with a noise density of 0.1 to Lena’s image and 0.3 density to resolution chart image. The proposed image encryption scheme has shown to be resistant to noise effects, as the recovered images remain recognizable even in the presence of noise interference.

### 4.9. Analysis of Data Loss 

**Figure 15:**
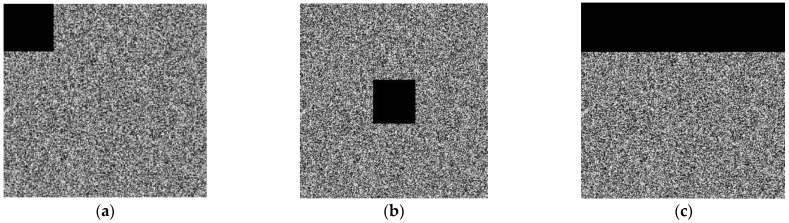

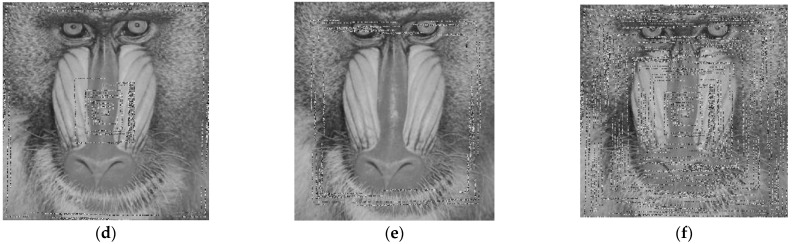
Data loss attacks. The encryption images with (**a**) 1/64, (**b**) 100/156 and (**c**) 1/256 occlusion, (**d**–**f**) are the corresponding decryption images.

This paper analyzes a specific ratio of the cipher image’s pixel value set to zero to test how the encryption method proposed in this research would affect data loss. After that, we tried to decrypt the modified cipher image. The results of the data loss attack are shown in Figure 15. As can be seen that our system is still able to generate an identifiable decrypted image even with a loss of more than 40% of pixels. As a result, this proposed algorithm is robust to data loss.

The cipher images Figure 15a–c of the baboon (256 × 256) with 1/64, 100/156, and 1/256 data loss, and the decrypted images Figure 15d–f of the baboon are shown under the cipher image. 

### 4.10. Time Complexity Analysis

The technique of measuring and analyzing the time necessary to carry out encryption operations on an image using a particular encryption method is known as computational time analysis in image encryption. Computational time analysis is used to evaluate the effectiveness and speed of the encryption algorithm as well as to identify any possible issues or potential areas for development. We calculated the encryption time for some images to calculate the performance of the proposed approach. On average, the encryption process takes 0.01568 s to finish. Some of them are listed below in the Table 8 and Table 9.

## 5. Conclusions

In this study, a new image encryption method has been introduced that is both secure and efficient without compromising on either aspect. We propose a new image encryption method that combines Intertwining Logistic Mapping (ILM) and Orbital Shift Pixel Scrambling Method (OSPSM). The proposed encryption method applies the OSPSM strategy to confuse image pixels. This method draws inspiration from the rotational motion of planets along their orbits, combined with chaotic sequences to disrupt the pixel positions of the image. The diffusion operation is carried out on the scrambled 2D matrix, generating a mask image using ILM. Then, the XOR operation is performed on the generated mask image and the scrambled 2D image matrix so the final ciphertext image is obtained. This process effectively destroys any intra-correlations among the pixels of the image, which helps to enhance the security of the encryption. Furthermore, the key generation method presented in this work using ILM is not solely dependent on the image itself but also on certain secret parameters. This highlights the importance of maintaining the confidentiality of these secret parameters to ensure the robustness and effectiveness of our proposed scheme.

## Figures and Tables

**Table 1:** Display of a comparison of the key space of our proposed scheme with other existing schemes.

Algorithms	Key Space
Ours	$1.16\times 10^{182}$
Ref. [28]	$10^{128}$
Ref. [34]	$2^{197}{\approx 2\times 10}^{59}$
Ref. [35]	$10^{90}$
Ref. [46]	$10^{105}$

**Table 2:** Original key parameter values.

Parameters	Values
(μ, *k*_1_, *k*_2_)	μ = 1.108021259307861, *k*_1_ = 2.143530845642090, *k*_2_ = 0.561828732490540
(*k*_3_, *x*_0_, *y*_0_)	*k*_3_ = 0.548855721950531, *x*_0_ = 1.093351840972900, *y*_0_ = 1.050179004669190,
(*z*_0_, *p*)	*z*_0_ = 1.139293670654297, *p* = 11

**Table 3:** Display of the rates of change between encrypted images resulting from the use of slightly modified keys.

Keys	NPCR		
	Lena	Baboon	Moon
${Key}_{1}(x^{\prime }=(x_{o}+10^{-15}))$	99.61	99.62	99.63
${Key}_{2}(y^{\prime }=(y_{o}+10^{-15}))$	99.63	99.61	99.60
${Key}_{3}(z^{\prime }=(z_{o}+10^{-15}))$	99.62	99.60	99.59
${Key}_{4}(\mu^{\prime }=(\mu_{o}+10^{-15}))$	99.60	99.59	99.59
${Key}_{5}(k_{1}=(k_{1}+10^{-15}))$	99.63	99.59	99.60
${Key}_{6}(k_{2}=(k_{2}+10^{-15}))$	99.61	99.62	99.64
${Key}_{7}(k_{3}=(k_{3}+10^{-15}))$	99.62	99.63	99.64

**Table 4:** Information entropy of plaintext images and ciphertext images.

Tested Images	Image Size	Plain Image	Proposed
Lena	256 × 256	7.4749	7.9970
Baboon	256 × 256	6.9729	7.9972
Airplane	256 × 256	6.4522	7.9970
Clock	256 × 256	6.7056	7.9970
Moon	256 × 256	6.7093	7.9974
Chemical plant	256 × 256	7.3424	7.9975

**Table 5:** Comparison of the proposed method with existing methods.

Tested Images	Image Size	Plain Image	Ref. [25]	Ref. [27]	Ref. [41]	Proposed
Lena	256 × 256	7.4749	7.9953	7.9957	7.9970	7.9970
Baboon	256 × 256	6.9729	\	7.9952	7.9971	7.9972
Airplane	256 × 256	6.4522	\	7.9952	7.9971	7.9970
Clock	256 × 256	6.7056	\	7.9955	7.9970	7.9970
Moon	256 × 256	6.7093	\	7.9956	7.9968	7.9974
Chemical Plant	256 × 256	7.3424	\	\	7.9969	7.9975

**Table 6:** NPCR and UACI values of our proposed method.

Test Images	NPCR	UACI
Lena	99.6338	33.2992
Airplane	99.5819	33.4625
Clock	99.6032	33.3568
Moon	99.6002	33.3076
Chemical plant	99.5965	33.4511

**Table 7:** The comparison of NPCR and UACI with other techniques.

Algorithms	Average NPCR	Average UACI
**Ref. [25]**	99.6091	33.4437
**Ref. [27]**	99.6282	33.2459
**Ref. [28]**	99.6000	33.4000
**Ref. [40]**	99.6110	33.2320

**Table 8:** Encryption time test.

Test Images	Size of Image	Ref. [27]	Ours
Lena	256 × 256	0.1842	0.1415
Baboon	256 × 256	0.1817	0.1586
Moon	256 × 256	0.1796	0.0156
Airplane	256 × 256	0.1834	0.1570
Clock	256 × 256	0.1856	0.1647

**Table 9:** Average values of encryption time comparison with existing methods.

Algorithms	Ref. [25]	Ref. [27]	Ref. [39]	Ours
**Execution time**	0.1830	4.0200	1.4800	0.1568

## Data Availability

Not applicable.
